# Hybrid Adsorbent from Construction and Mollusk Shell Wastes for Model Cationic Dye Removal: Kinetic Performance, Thermodynamic Insights, and Fenton Regenerability

**DOI:** 10.1021/acsomega.6c04839

**Published:** 2026-08-18

**Authors:** Danilo Henrique da Silva Santos, Larissa Farias Queiroz, Luiz Daniel Silva Neto, Keven Ewerton dos Santos, Denio Deivison Cerqueira Silva das Neves, Daniel Pinto Fernandes, Lucas Meili

**Affiliations:** † Laboratory of Processes Applied to Water Treatment, 28112 Technology Center of Federal University of Alagoas, Av. Lourival Melo Mota, s/n, Campus A.C. Simões, Tabuleiro do Martins, Maceió, AL 57072-970, Brazil; ‡ Bioenergy and Energy Efficiency Laboratory, Institute for Technological Research, São Paulo, São Paulo 05508-901, Brazil

## Abstract

Dye-containing effluents remain a concern in wastewater treatment because many synthetic dyes are persistent, chemically stable, and potentially toxic. In parallel, the construction industry, although closely linked to economic and social development, generates large amounts of construction and demolition waste (CDW), which often lacks adequate disposal. Mollusk shells are another locally abundant residue with limited reuse. Converting these abundant residues into low-cost adsorbents offers a promising strategy for wastewater treatment and waste valorization. This study evaluates the efficiency of an innovative hybrid adsorbent composed of CDW and *Mytella charruana* (MC) shells for removing methylene blue (MB) from aqueous solution. The composite CDW-MC (75:25, m m^–1^) produced a synergistic effect, increasing the adsorption capacity to approximately 8.0 mg g^–1^ under initial screening conditions, a gain of about 14% and 37% relative to CDW alone (7.00 mg g^–1^) and MC alone (5.85 mg g^–1^), respectively. Under equilibrium conditions, the Toth model estimated a maximum adsorption capacity of 20.83 mg g^–1^ at 60 °C for the CDW-MC composite. Characterization by scanning electron microscopy (SEM), Fourier transform infrared spectroscopy (FTIR), X-ray diffraction (XRD), X-ray fluorescence (XRF), Brunauer–Emmett–Teller (BET) analysis, and point of zero charge (pH_PZC_) indicated that the composite had a heterogeneous surface containing carbonate-, silicate-, and metal oxide-related sites. The adsorption process was found to be spontaneous, exothermic, and predominantly physical in nature, with a positive entropic contribution. Regeneration of the saturated adsorbent via the Fenton reaction, optimized through a factorial experimental design, demonstrated that Fe^2+^ concentration was the decisive factor governing process efficiency, maintaining regeneration performance above 70% over five successive cycles. Overall, these results show that the CDW-MC composite offers a technically feasible and environmentally sustainable route for treating dye-contaminated wastewater while promoting waste valorization, adsorbent reuse, and circular-economy principles.

## Graphical abstract

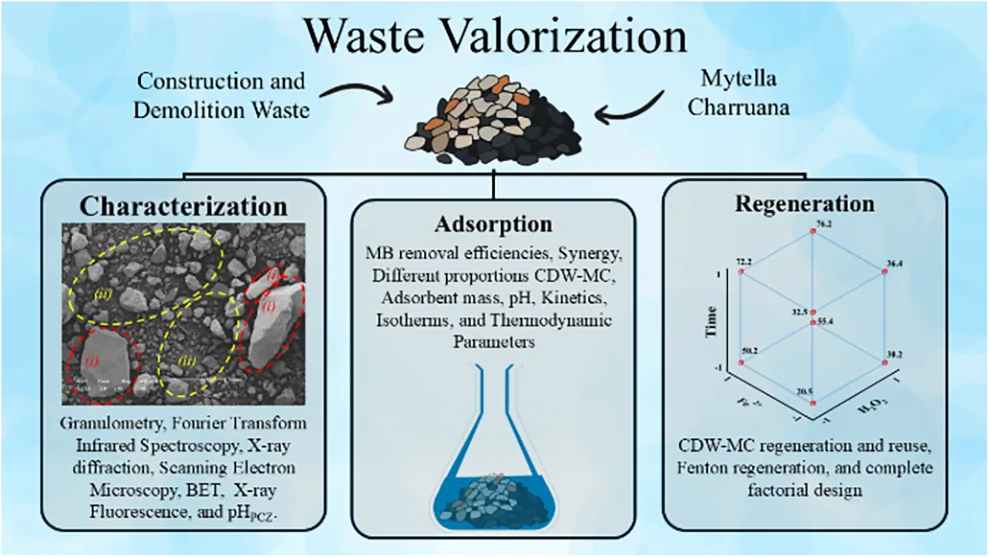

## 1 Introduction

Rapid industrialization and technological advances have been identified as major drivers of water-body contamination by chemical compounds.^1,2^ Among the various sources of pollution, the discharge of dye-containing effluents represents one of the most severe environmental threats.^3^ The complex aromatic structures of synthetic dyes confer high molecular stability, promoting ionization and chemical–microbiological oxidation in wastewater, and generating potentially hazardous byproducts.^4^ Furthermore, many of these compounds exhibit toxicity, mutagenicity, and carcinogenic potential, posing significant risks to both aquatic ecosystems and human health.^4,5^ To address this issue, several dye removal techniques have been reported in the literature, including adsorption,^6,7^ filtration,^8,9^ oxidation,^10,11^ biological treatment,^12,13^ coagulation,^14,15^ and flocculation.^16,17^ However, many of these methods are associated with notable limitations, including sludge generation, high operating costs, slow kinetics, and a high consumption of chemical reagents.^18^

Among the available techniques, adsorption is the most widely used method for treating dye-containing effluents.^5^ This approach offers considerable advantages, including simplicity of operation, low cost, and resistance to aggressive chemical agents.^19,20^ Moreover, spent adsorbents can often be regenerated and reused, minimizing the generation of harmful byproducts and reducing overall operational costs.^21,22^ The efficiency of the adsorption process is governed by the surface characteristics of the adsorbent material and its specific interactions with the target pollutants.^19,23^ Although commercial adsorbents, such as zeolites, activated alumina, silica gel, activated carbon, and synthetic polymers, are widely employed,^24^ their large-scale application remains constrained by high production and processing costs.^18,25^

Several studies have highlighted the use of low-cost materials with high adsorbent capacity, derived from natural sources or industrial, agricultural, and forestry residues, for the removal of dyes from wastewater.^21^ Construction and demolition waste (CDW) corresponds to 30–50% of all solid waste generated by the construction industry, a key indicator of socioeconomic development. These materials constitute one of the most significant urban challenges, as most of them are not adequately disposed of.^26^ Although recycling and reuse in the construction sector are the predominant practices for CDW management, exploring alternative uses, such as transforming these wastes into adsorbents for the removal of aquatic pollutants, is a promising strategy to increase the industry’s sustainability.^27–29^ Several studies have demonstrated the potential of CDW as an adsorbent in wastewater treatment. Vallini et al.^28^ evaluated the use of building waste to remove metals from industrial effluents, achieving efficiencies greater than 90%. Song et al.^30^ produced chabatiza from CDW, achieving a maximum MB adsorption capacity of 129.18 mg g^–1^ at 298 K. dos Reis et al.^31^ used inorganic CDW sludge for phosphate sorption in aqueous solution, registering a maximum capacity of 57.64 mg g^–1^.

Another growing concern is the waste from *Mytella charruana* (MC), a byproduct generated on a large scale due to its widespread consumption.^32–34^ In view of the environmental impacts of improper disposal of this material, strategies have been developed to add environmental and economic value to the shells. Studies demonstrate the potential of these shells as absorbents for removing the triarylmethane biocide^32^ and rifampicin.^34^ In addition, more recent investigations also evaluate their reuse as a raw material in the synthesis of layered double hydroxides,^35^ expanding their applications in environmental technologies.^36^

The development of composite materials that combine the structural functionality and surface properties of their precursors offers multifunctionality and can significantly enhance adsorption performance through synergistic interactions between their components.^37^ In this context, the present study investigates the potential of a hybrid adsorbent produced from CDW and processed MC shells for the removal of methylene blue (MB) from an aqueous solution. Beyond its technical merits, the production of this material carries meaningful environmental and economic implications, contributing to the mitigation of liabilities associated with the improper disposal of both wastes and supporting the principles of a circular economy.

## 2 Materials and Methods

### 2.1 Adsorbent Preparation and Characterization

The raw materials used to produce the adsorbents were obtained in the municipality of Maceió, Alagoas, Brazil. The CDW was preprocessed to remove noninorganic components, such as plastics and wood, yielding a fraction predominantly composed of concrete, bricks, ceramics, and mortar. Subsequently, the CDW and MC samples were washed exclusively with distilled water (no detergent/soap) to remove adhered surface impurities, and then oven-dried at 30 °C for 24 h. Then, they were crushed and granulometrically classified by sieving with standard test sieves (nominal openings of 0.84, 0.59, 0.50, 0.42, and 0.29 mm) into four particle-size fractions: <0.29, 0.29–0.42, 0.50–0.59, and 0.59–0.84 mm.

The functional groups on the surfaces of the studied materials were determined by Fourier transform infrared spectroscopy (FTIR, Shimadzu IR PRESTIGE-21) using an attenuated total reflection (ATR) accessory. The spectra were obtained in the 4000–400 cm^–1^ region. A baseline was subtracted using anchor points placed in band-free regions (Peak Analyzer, OriginPro 2018), followed by Savitzky–Golay smoothing (21 points, third-order polynomial). The mineralogical composition of CDW and MC materials was analyzed by X-ray diffraction (XRD, Shimadzu XRD-6000). The XRD standards were obtained at 40 kV and 30 mA, over 5–80° with a pitch of 0.02°. The divergence and scattering slits used were 1°, and the reception slits were 0.30 mm, and the Cu Kα radiation was used. Crystalline phases were identified using the X’Pert HighScore software with reference to standard crystallographic databases, and diffractograms are presented without additional smoothing or background subtraction. The morphological characterization of the CDW was investigated using scanning electron microscopy (SEM, JEOL JSM-6610), operated at an accelerating voltage of 15 kV and a working distance of 16 mm, using a secondary electron (SE) detector at 45× magnification; the samples were previously sputter-coated for 5 min with a platinum target and 5 min with a gold target, both at 10 mA. The elemental composition was determined by energy-dispersive X-ray fluorescence (EDXRF, Shimadzu EDX-800HS) equipped with a rhodium X-ray tube, operated under vacuum with a 10 mm collimator. The oxide contents were quantified from the measured elemental intensities using the standardless fundamental parameters method implemented in the instrument software. The textural properties were evaluated by N_2_ adsorption/desorption at 77 K (Quantachrome NOVA, NovaWin software v11.0). The samples were pretreated at 250 °C for 24 h. The specific surface area was determined using the BET method, and the total pore volume and average pore diameter were obtained from the adsorption/desorption isotherm. The surface chemistry of the materials was characterized by determining the point of zero charge (pH_PZC_) using the methodology adapted from Nethaji et al.^38^ For this, suspensions of the adsorbents (0.15 g) were prepared in 0.01 mol L^–1^ NaCl solutions, with the pH adjusted to 2–12. The suspensions were shaken at 150 rpm for 48 h. After this period, the final pH was measured, and the pH variation was plotted as a function of the final pH to determine the pH_PZC_.

### 2.2 Adsorption Tests

In the adsorption experiments, the impact of the particle-size fraction (0.42–0.29 mm) of the precursor materials, CDW and MC, was initially evaluated to identify the most suitable fraction for use as an adsorbent. In sequence, the effects of the composition of the CDW-MC adsorbent (100:0, 75:25, 50:50, 25:75, and 0:100 m m^–1^), the adsorbent mass (0.05–0.4 g), and the solution pH (3–11) on the efficiency of MB removal were investigated. The pH control was performed by adding 0.1 mol L^–1^ HCl or NaOH solutions. All experiments were carried out on a shaker table operating at 150 rpm and 30 °C. The MB concentration was quantified using a UV–vis spectrophotometer (Shimadzu UV-1280) at 665 nm. The removal efficiency and adsorption capacity were calculated using eqs 1–3.

1 $$R=100\times \frac{\left(C_{i}-C_{e}\right)}{C_{o}}$$

2 $$q_{t}=\frac{\left(C_{i}-C_{t}\right)\times V}{m}$$

3 $$q_{e}=\frac{\left(C_{i}-C_{e}\right)\times V}{m}$$

where is the removal percentage *R* (%); *q*_*e*_ and *q*_*t*_ are the amount of MB adsorbed by the adsorbent (mg g^–1^) at equilibrium and *t* time (min), respectively; *C*_*i*_, *C*_*t*_, and *C*_*e*_ are the initial, *t* time, and equilibrium concentrations (mg L^–1^), respectively; *V* is the volume of the solution (L); and *m* is the mass of the adsorbent (g).

### 2.3 Kinetics, Isotherms, and Thermodynamics

The kinetic study was performed using 0.1 g of CDW-MC and 20 mL of MB (pH 9 and 50 mg L^–1^). The experiments were carried out in a finite bath (150 rpm) at room temperature (30 °C). Samples were collected at 5, 15, 30, 45, 60, 120, 180, 240, and 300 min. The kinetic data were evaluated and fitted to the pseudo-first-order (PFO) (eq S1), pseudo-second-order (PSO) (eq S2), Elovich (eq S3), intraparticle diffusion (eq S4), and Boyd (eqs S5, S6, and S7) models.^39^

Adsorption isotherms were performed using 0.1 g of CDW-MC and 20 mL of MB (pH 9). The isotherms were performed at concentrations of 5, 10, 20, 30, 40, 50, 100, 200, 300, 400, and 500 mg L^–1^ at temperatures of 30, 40, 50, and 60 °C. The data obtained were evaluated and fitted to the Langmuir (eq S8), Freundlich (eq S9), Redlich–Peterson (eq S10), Sips (eq S11), and Toth (eq S12) models.^40–45^

Gibbs’ free energy thermodynamic parameters (Δ*G°*), enthalpy variation (Δ*H°*), and the entropy variation (Δ*S°*) of the system are determined by the best isothermal adjustment.^46,47^ Assuming that the variations in enthalpy and entropy are independent of temperature, the relationship between Δ*G°*, Δ*H°*, and Δ*S°* is expressed through eqs S13 and S14. Through the graph generated by the van’t Hoff eq (eq S15), the values of Δ*H°* and Δ*S°*.^48,49^

The evaluation of the quality of the adjustments of the mathematical equations was carried out based on the coefficient of determination (*R* ^2^) (eq S16), chi-square (*x* ^2^) (eq S17), average relative error (ARE) (eq S18), and the Akaike information criterion (AIC) (eqs S19 and S20).^50,51^

### 2.4 Regeneration of Saturated CDW-MC and Statistical Analysis

The Fenton reaction was used to regenerate the CDW-MC composite material saturated with MB. The influence of operational variables, H_2_O_2_ concentration (*H*), Fe^2+^ concentration (*F*), and contact time (*T*), on regeneration efficiency was evaluated using a 2^3^ complete factorial design. The experimental ranges and coded levels of the independent variables are presented in Table 1.

**Table 1:** Independent Variables and Coded Levels Used in the 2^3^ Factorial Design for the Fenton Regeneration of MB-Saturated CDW-MC

		**levels**	
**code**	**parameters**	**–1**	**+1**
*H*	H_2_O_2_ (mmol L^–1^)	100	500
*F*	Fe^2+^ (mmol L^–1^)	0	0.5
*T*	time (h)	1	4

The significance of the operational parameters in the regeneration of the adsorbent material was determined by analysis of variance (ANOVA). The experimental data were analyzed using Statistica software, version 13. The response variable adopted was the percentage of regeneration (eq 4).^52^

4 $$R_{r}=\frac{q_{r}}{q}\times 100$$

where *R*_*r*_ is regeneration (%), *q*_*r*_ is the adsorption capacity measured in equilibrium after resorption with regenerated adsorbent, and *q* is the initial loading of the adsorbate in equilibrium (mg g^–1^).

## 3 Results and Discussion

### 3.1 Material Characterization

Figure 1a shows the diffractograms of the CDW, the MC, and the CDW-MC composite used in the experiments, all of which exhibit characteristic reflections. Figure 1a confirms the carbonaceous nature of MC and identifies aragonite as its predominant crystalline phase. The proportion of aragonite to calcite in the microstructure and crystal orientation of mollusk shells varies according to species and region.^32,34^ The result for CDW indicates a crystalline mineralogical composition composed of quartz (SiO_2_), calcite (CaCO_3_), and calcium oxide (CaO, Ca­(OH)_2_), typical elements of samples containing sand, concrete, and mortar.^31,53^ Comparing the XRD patterns, the full width at half-maximum (fwhm) of the main diffraction peaks of CDW and MC are comparable, indicating that both precursor materials are similarly well-crystallized. In the composite, the reflections exhibit reduced relative intensity compared to CDW and MC due to the dilution resulting from the physical mixture of the materials. Since the composite preparation did not involve thermal or chemical treatment, no new crystalline phase was formed. Minor differences in peak profiles were also observed between duplicate diffractograms of the CDW-MC composite, attributed to the mineralogical heterogeneity inherent to CDW.^27–29^ The combination of carbonaceous material and inorganic oxides in the composite can enhance its potential as an adsorbent by providing additional active sites, thereby improving adsorptive performance in aqueous solutions.^31,53^

**Figure 1:**
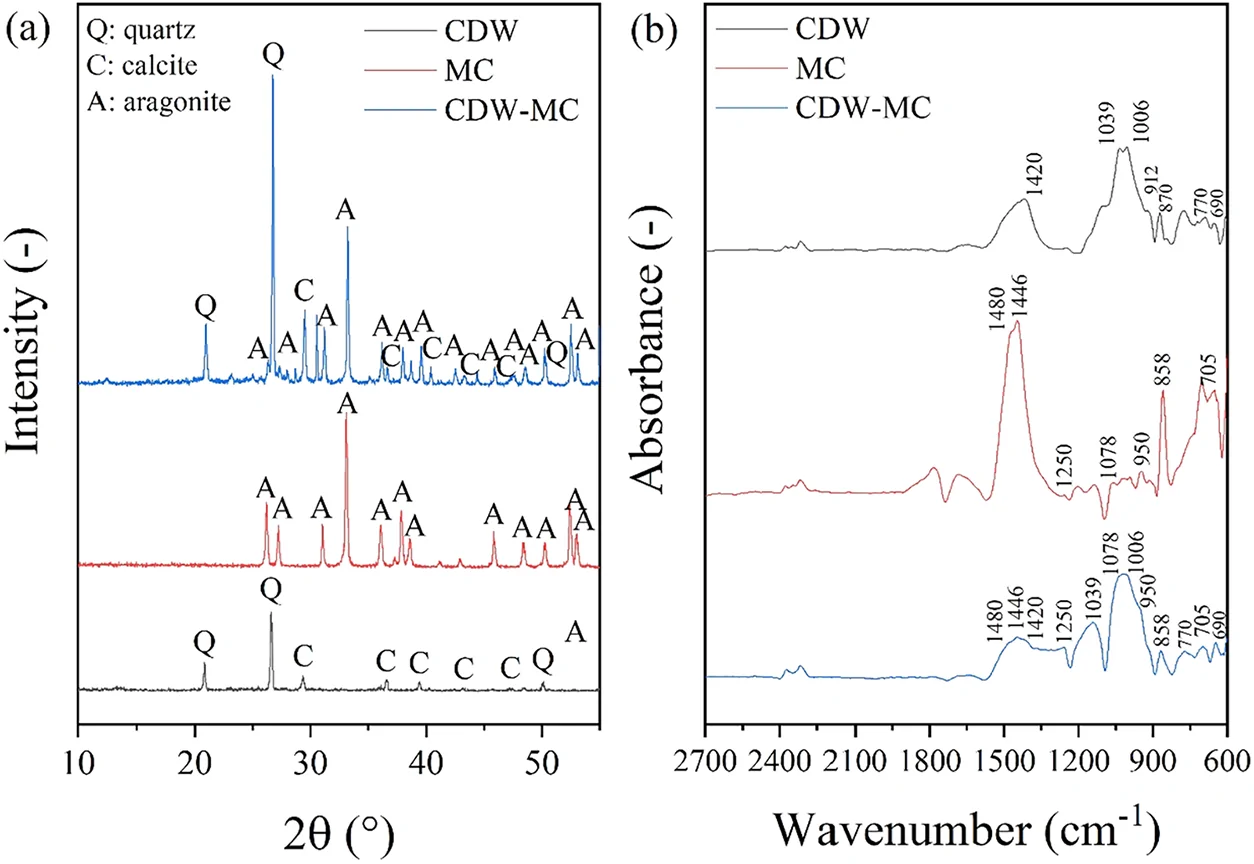
Physicochemical characterization of CDW, MC, and the CDW-MC composite: (a) XRD patterns showing the main crystalline phases, including quartz (Q), calcite (C), and aragonite (A). (b) ATR-FTIR spectra showing the main functional groups associated with silicates, carbonates, and oxide-containing phases.

Figure 1b shows the infrared absorption spectra. The MC spectrum has characteristic bands typical of mollusk shells. The vibration modes at 705, 858, 950, 1250, 1446, and 1480 cm^–1^ confirm the presence of the carbonate group in the material. The band at 705 cm^–1^ corresponds to angular deformations in the O–C–O connection plane. The bands at 858 and 950 cm^–1^ refer to angular deformations of the connecting plane of the CO_3_^2–^, identifying the presence of aragonite and calcite in the material and corroborating the result of XRD.^54^ The band at 1078 cm^–1^ is attributed to symmetric elongation, and the band at 1446 cm^–1^ is due to asymmetric stretching of the C–O bond.^33^ The CDW spectrum shows characteristic bands of silica oxide in the form of quartz (SiO_2_) and calcite (CaCO_3_), which are typical of concrete- and mortar-containing samples.^53^ The spectra observed between 1400 and 1500 cm^–1^ are associated with the carbonate group and C=O-type bonds.^55^ The peak at 1039 cm^–1^ corresponds to the C–O stretching vibration.^56^ Peaks at 870 and 1420 cm^–1^ are also characteristic of the carbonate group.^53^ The peak around 1000 cm^–1^ is attributed to the asymmetric vibration of Si–O–Si and Si–O–Al or to the elongation vibration of the SiO_4_ tetrahedron.^30^ Vibrations at 770 and 690 cm^–1^ represent the existence of Si–O bonds in the material.

Figure 2 shows a scanning electron microscopy image that reveals the morphological differences among the materials. CDW shows a disordered agglomeration of particles, with irregular fragments, varying sizes, rounded angles, and a rough, inhomogeneous surface (Figure 2a), whereas MC displays a more cohesive, regular, and elongated morphology (Figure 2b). Crystallinity was assessed independently from morphology based on the XRD peak widths discussed above (Figure 1a), which indicated comparable crystallinity between the two precursor materials. Figure 2c presents a micrograph of the composite, in which the structures of the CDW and the MC are observed.

**Figure 2:**
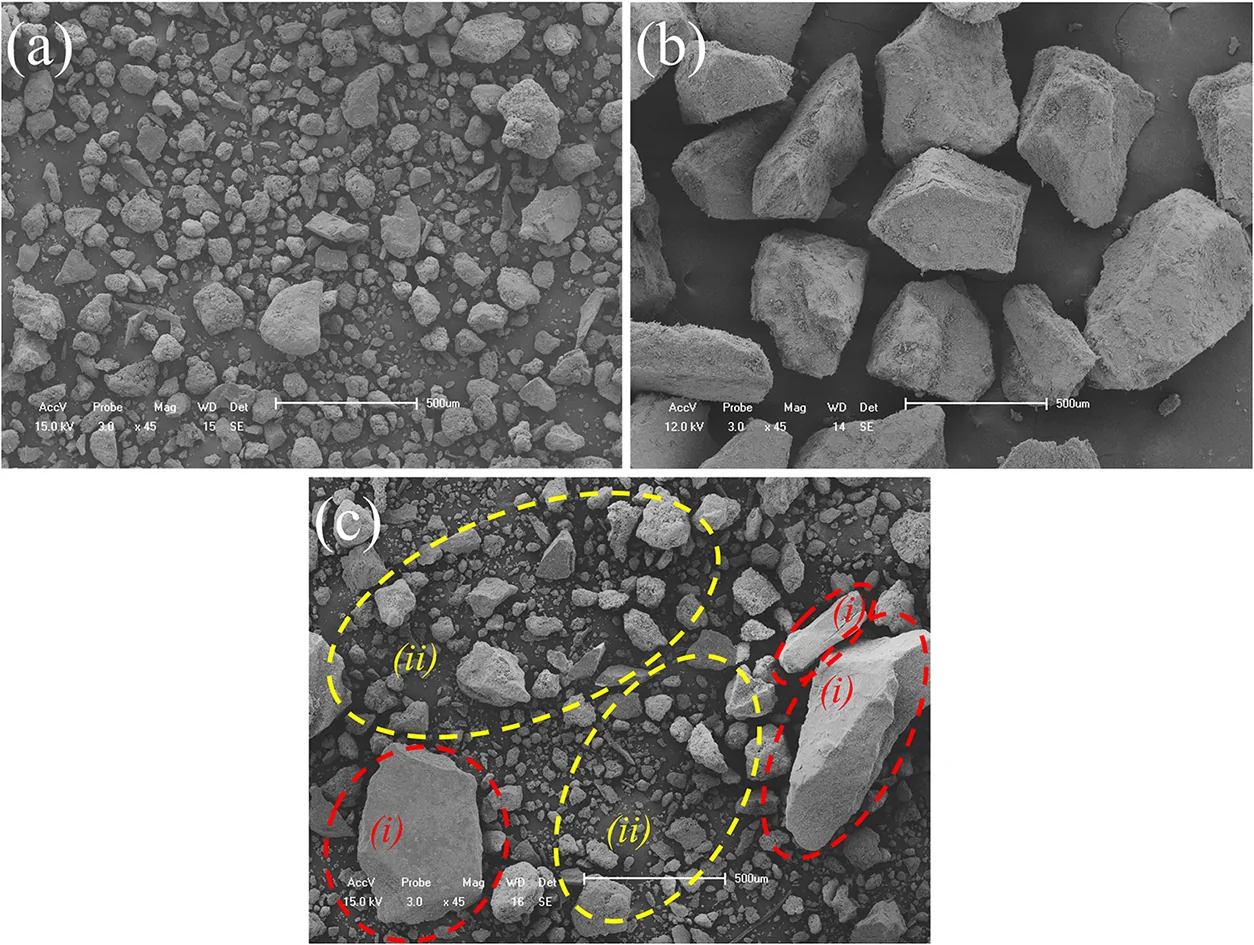
Scanning electron microscopy images showing the surface morphology of the precursor materials and composite adsorbent: (a) CDW, characterized by irregular particles and a rough, heterogeneous surface; (b) MC, showing a more cohesive and crystalline structure; and (c) CDW-MC composite, in which MC particles (i) and CDW particles (ii) can be distinguished.

Table 2 shows the elemental composition of the CDW-MC composite. These values represent an oxide-normalized elemental composition rather than a direct identification of discrete oxide phases. According to XRF, Si, Ca, Al, Fe, Sc, K, S, Mg, Sr, Mn, and Zn were identified as the main elemental components of the material, with SiO_2_ (43.00%) and CaO (28.69%) as the most abundant. These results are consistent with the crystalline phases independently identified by XRD (Figure 1a) (quartz (SiO_2_), calcite (CaCO_3_), and calcium oxide/hydroxide (CaO, Ca­(OH)_2_)), which confirm the actual mineral phases present rather than the individual oxides reported by XRF. Given that the CDW is derived from mixtures of concrete, cement, and aggregates, mixed metallic phases may also be present. Consequently, the concentrations of these constituents can vary depending on the phase proportions and the construction requirements of the region of origin.^26,57^ The Fenton-type regeneration reaction is favored by the presence of these catalytic oxides in the material itself.^58^

**Table 2:** Elemental Oxide Composition of the CDW-MC Determined by X-ray Fluorescence

**CDW-MC composition (%)**										
SiO_2_	CaO	Al_2_O_3_	Fe_2_O_3_	Sc_2_O_3_	K_2_O	SO_3_	MgO	SrO	MnO	ZnO
43.00	28.69	14.20	5.29	4.20	1.88	1.34	0.99	0.26	0.11	0.03

Figure 3 shows the N_2_ adsorption/desorption curve for the CDW-MC composite. The isotherm obtained is of type IV, with type H3 hysteresis. Type IV isotherms are characteristic of mesoporous solids, with an evaporation process distinct from condensation. Hysteresis loops of the H3 type are attributed to materials with plate-like particles, such as some clays, as well as to materials with macropores that were not filled during condensation.^30,59^ The absence of hysteresis does not imply the absence of porosity, as certain pore shapes can yield identical adsorption and desorption isotherms.^59^ The results for surface area and total pore volume were 12.463 m^2^ g^–1^ and 0.046 cm^3^ g^–1^, respectively. In addition to the CDW naturally presenting structural variations by region of acquisition, differences in Brunauer–Emmett–Teller (BET) surface area may reflect differences in particle size, which is not strictly controlled during the crushing process.^26^

**Figure 3:**
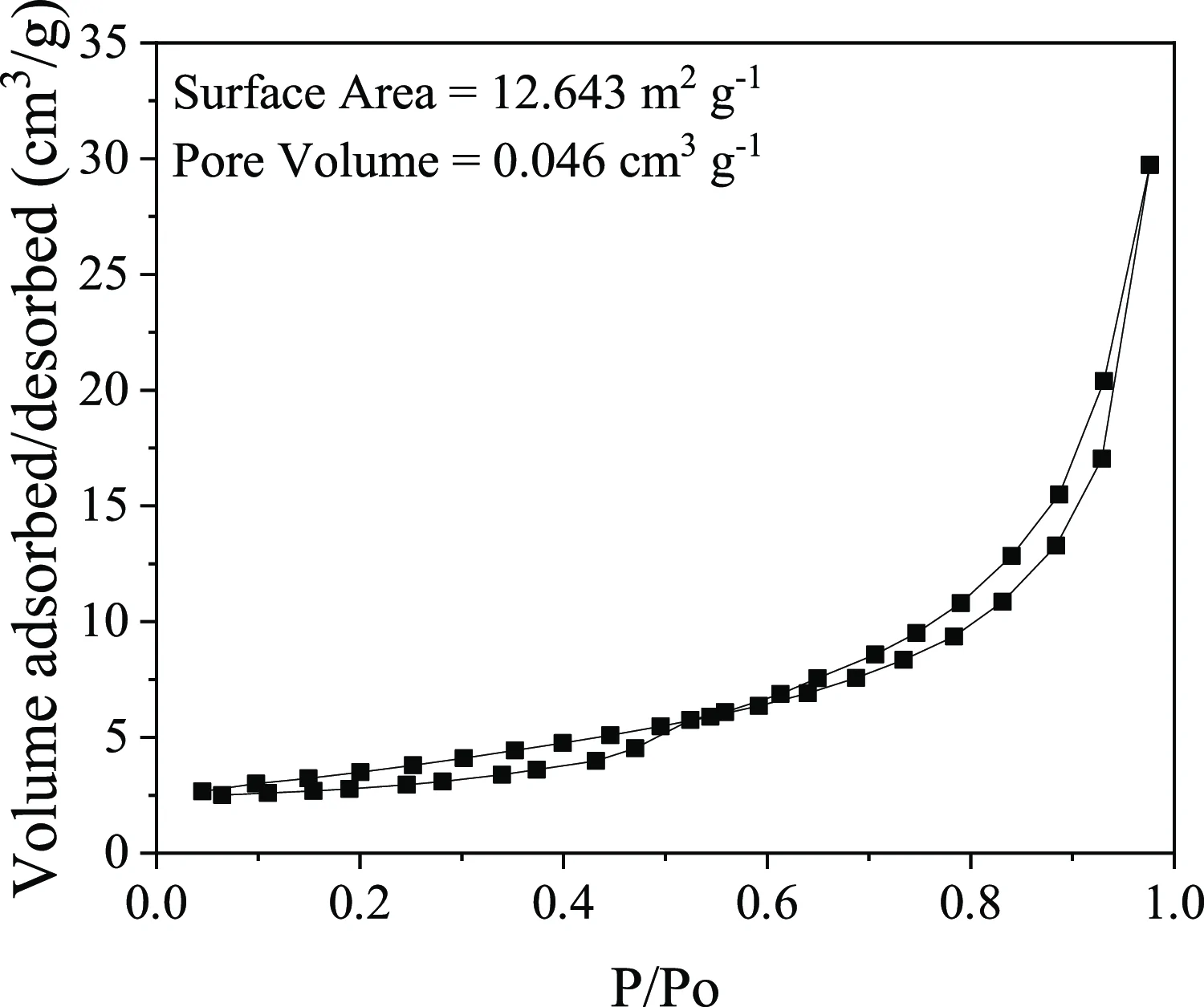
N_2_ adsorption–desorption isotherm of the CDW-MC composite.

### 3.2 Adsorption Assays

The MB removal efficiencies of the CDW and MC adsorbents showed an inverse correlation with mean particle diameter (Figure S1), with adsorption efficiency increasing progressively as particle size decreased. For the smallest particle-size fractions, the maximum adsorption capacities reached 7.00 mg g^–1^ for CDW and 5.85 mg g^–1^ for MC. This behavior can be attributed to the increase in specific surface area and the reduction in internal diffusion resistance within the pores, which favor mass transport and adsorption.^60,61^ CDW exhibited superior adsorption performance, which can be attributed to multiple possible interactions, including chemisorption, electrostatic interactions, and ion exchange, between the adsorbate and the highly heterogeneous composition of CDW.^26^ Although adsorption capacity increased with decreasing particle size, the intermediate 0.29–0.42 mm fraction was selected for all subsequent experiments as a compromise between performance and practical handling. Beyond the difficulties of separating very fine particles from solution and the generation of fines, the sub-0.29 mm fraction is inherently heterogeneous, encompassing a wide range of particle sizes rather than a narrow, well-defined range. The 0.29–0.42 mm fraction thus offers a more suitable balance between adsorption efficiency and the powder-handling properties required for practical, scalable application.^62,63^

This superior adsorption effect of CDW on MC was also observed in the assays performed with the CDW-MC composite in different proportions (Figure 4a). MB was effectively removed in the different compositions (CDW-MC w w^–1^) of mixed adsorbents produced. However, MC’s contribution to these results is less significant, with dye removal decreasing as MC increases in the composite. The combination of CDW and MC will increase the number of active groups on the adsorbent surface, as observed in the material characterization results (Figure 1b), thereby improving the adsorption efficiency of MB. Synergy indices (ξ) were 1.16 and 1.60 in relation to CDW and MC, respectively, according to eq 5.^58^ The 75:25 CDW-MC mixture (m m^–1^) showed a higher adsorption capacity, approximately 8.0 mg g^–1^, exceeding the values obtained for the isolated precursor materials (7.00 and 5.85 mg g^–1^ for CDW and MC, respectively), evidencing the synergy between the components and the formation of more active adsorption sites.

5 $$\xi =\frac{q_{\mathrm{composite}}}{q_{\mathrm{single}}}$$

where *q*_composite_ represents the removal efficiency of the combined materials and *q*_single_ represents the adsorption removal efficiency of the isolated materials.

**Figure 4:**
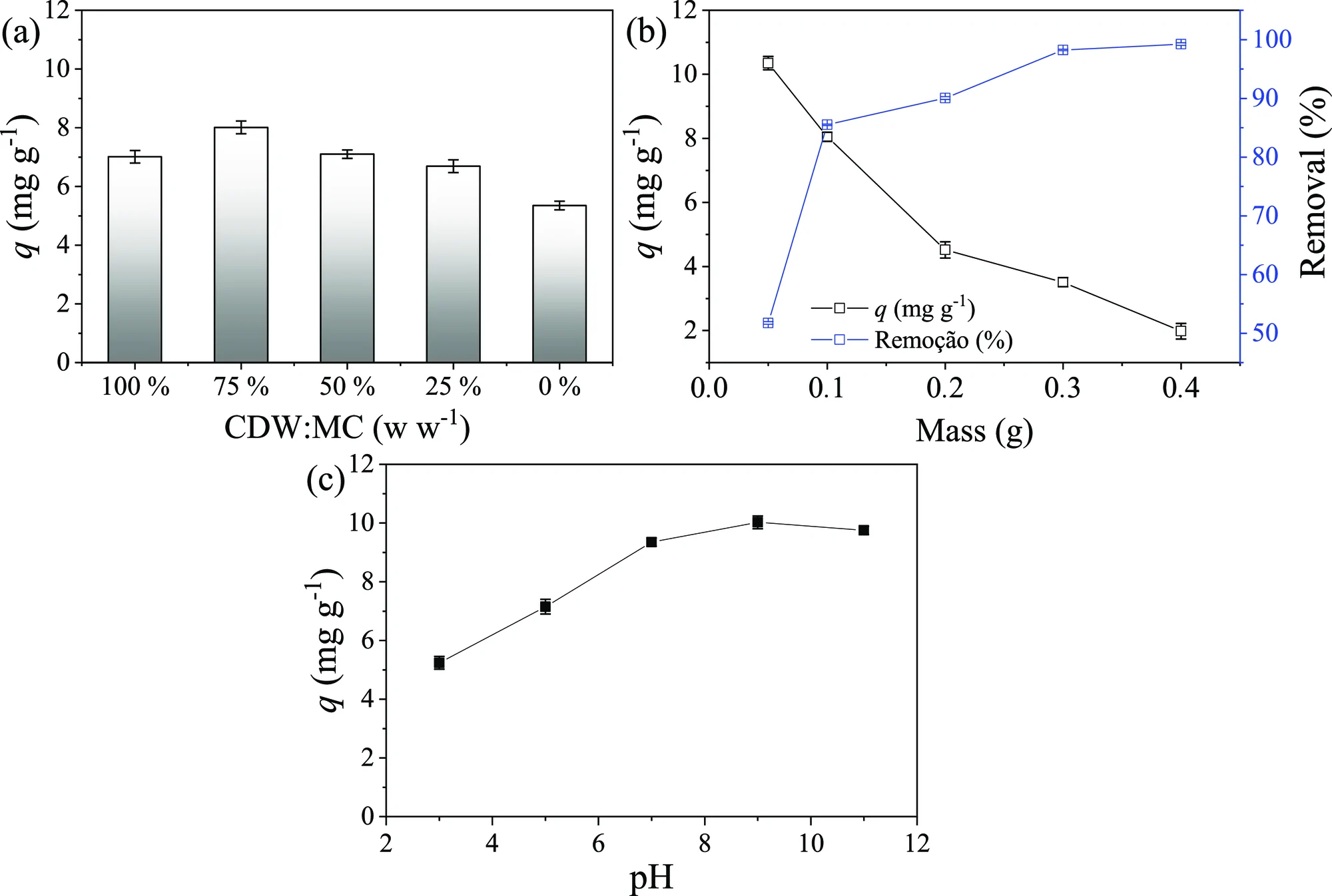
Effects of operational parameters on MB adsorption by the CDW-MC composite: (a) influence of the CDW:MC mass ratio on adsorption capacity; (b) effect of adsorbent mass on MB adsorption capacity and removal efficiency; and (c) effect of initial solution pH on MB adsorption capacity and removal efficiency.

Increasing the amount of adsorbent enhances the availability of active sites for adsorbent–adsorbate interactions,^64^ thereby improving MB removal efficiency (Figure 4b). Increasing the mass from 0.05 to 0.2 g increased removal from 50.15 to 90.04%, approximately 40%. For adsorbent dosages greater than 0.2 g, the gain is less significant: from 90.04 to 98.24% when increasing from 0.2 to 0.3 g and then becoming practically stable between 0.3 and 0.4 g. Above 0.2 g, the system may reach equilibrium, leaving free active sites that do not contribute to the adsorption process.^65^ This excess of unsaturated active sites reduces the amount of adsorbate retained per unit mass of adsorbent,^66^ leading to an inverse effect on adsorption capacity, which decreases from 10 to 2 mg g^–1^ as the adsorbent mass increases from 0.05 to 4 g, respectively.

The pH of the solution significantly influenced the adsorption efficiency, with the adsorption capacity increasing by approximately 5.0 mg g^–1^ at pH 3 to approximately 10 mg g^–1^ at pH 9, remaining practically constant above pH 9 (Figure 4c). This behavior reflects the intensification of electrostatic interactions.^67^ As it is a cationic dye, MB will be removed more efficiently from negatively charged surfaces.^68^ The zero charge potential (pH_PZC_ = 10,29) confirms that at pH > pH_PZC_, the surface of the adsorbent is negative, favoring the removal of MB (Figure S2). When pH < pH_PZC_, the MB removal efficiency is suppressed by two important phenomena: electrostatic repulsion between the positive charge of the adsorbent surface and the MB cations coexisting under these conditions, and reduction of the active sites available for adsorption by competition between H^+^ and MB molecules.^69^ However, high values of *q* still occur in pH < pH_PZC_, indicating that other mechanisms, such as hydrogen bonds with amino and hydroxyl groups, and π–π interactions between the aromatic structures of the composite CDW-MC and MB, also contribute to adsorption.^70^

### 3.3 Adsorption Kinetics

The adsorption capacity of CDW-MC over time is shown in Figure 5. The adjustment of the PFO, PSO, and Elovich models to the experimental data yielded a satisfactory fit (Figure 5a). Two distinct stages were observed during the adsorption process: an initial rapid uptake stage, in which the adsorption capacity reached 8.2 mg g^–1^ within 45 min, followed by a slower stage approaching kinetic equilibrium, with a capacity of approximately 9.3 mg g^–1^ after 2 h of contact. The high initial adsorption rate can be attributed to the availability of active sites and the high adsorbate concentration in solution, both of which decrease as adsorption proceeds.^71^ Table 3 presents the kinetic parameters obtained from the fitted models, demonstrating that the PSO provided the best fit to the experimental data. The PSO model assumes that the occupancy rate of active sites for adsorption is proportional to the total number of free sites and does not specify any mechanism for adsorbate–adsorbent interactions.^72–74^

**Figure 5:**
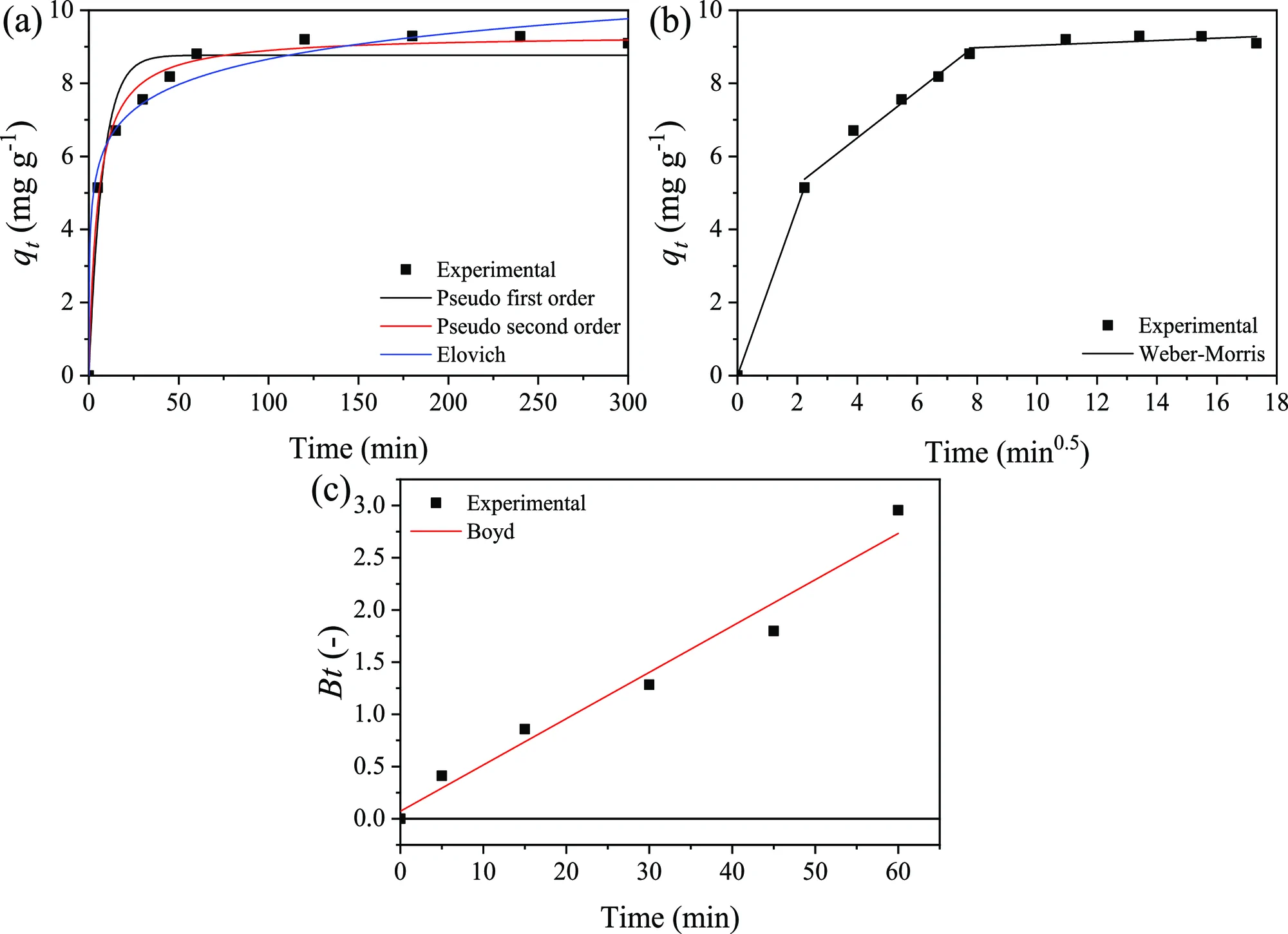
Adsorption kinetics of methylene blue onto the CDW-MC composite: (a) experimental data fitted to the pseudo-first-order (PFO), pseudo-second-order (PSO), and Elovich kinetic models; (b) intraparticle diffusion model; and (c) Boyd model.

**Table 3:** Kinetic Parameters Obtained for Methylene Blue onto the CDW-MC Composite Using the Pseudo-First-Order (PFO), Pseudo-Second-Order (PSO), and Elovich Models^a^

**model**	**parameters**	**CDW-MC**
PFO	*q*_*e*_ (mg g^–1^)	8.7694
	*k*_1_ (min^–1^)	0.1308
	*R* ^2^	0.9502
	*x* ^2^	0.4744
	ARE	0.7116
	AIC	–1.772
PSO	*q*_*e*_ (L g^–1^)	9.3362
	*k*_2_ (min^–1^)	0.0218
	*R* ^2^	0.9905
	*x* ^2^	0.0903
	ARE	0.3157
	AIC	–16.701
Elovich	α_*E*_ (mg g^–1^ min^–1^)	55.325
	β_*E*_ (g mg^–1^)	0.9942
	*R* ^2^	0.9809
	*x* ^2^	0.1818
	ARE	0.3783
	AIC	–10.404

a *q*_*e*_: equilibrium adsorption capacity; *k*_1_: PFO rate constant; *k*_2_: PSO rate constant; *α*_*E*_: initial adsorption rate; *β*_*E*_: Elovich desorption constant; *R* ^2^: coefficient of determination; *x* ^2^: chi-square statistic; *ARE*: average relative error; *AIC*: Akaike information criterion.

The Weber–Morris model (1963) was used to evaluate whether intraparticle diffusion is the rate-limiting step in the adsorption process, as shown in Figure 5b.^75^ The values of the intraparticle diffusion coefficients and diffusion resistance are shown in Table 4. The results indicate the existence of three linear segments, suggesting that multiple steps control the adsorption mechanism. The first segment demonstrates the boundary-layer effect, indicating a significant resistance to external mass transfer at the beginning of the process. The second segment represents a phase of progressive adsorption, governed by intraparticle diffusion. The third segment corresponds to the system’s equilibrium, at which point intraparticle diffusion begins to decrease, possibly due to a reduction in the methylene blue concentration.^76^ The increase in the value of *C*_*I*_ over the time of adsorption reflects an intensification of the boundary layer and, consequently, of the diffusion resistance. This effect is also evident in the variation of the intraparticle diffusion coefficient, which reduces as the dye is adsorbed, indicating a lower intraparticle diffusion as the process progresses. Thus, the Weber–Morris model suggests that both mass transfer and intraparticle diffusion play key roles in the adsorption mechanism of methylene blue.^77–79^ The Boyd model was used to verify whether the control of the adsorption process follows the pattern identified by the Weber–Morris model. The profile obtained, illustrated in Figure 5c, shows that the experimental data do not pass through the origin, indicating that adsorption is governed by more than one mechanism. Thus, the results of the intraparticle diffusion model are reaffirmed, strengthening the understanding of the phenomena involved in adsorption.^80,81^ These results corroborate the best fit obtained by the PSO kinetic model for the experimental data.

**Table 4:** Intraparticle Diffusion Parameters for Methylene Blue onto the CDW-MC Composite, Obtained Using the Weber–Morris Model^a^

**segment**	***k***_***d***_ **(mg g** ^**–1**^ **min** ^**–0,5**^ **)**	***C***_***I***_ **(mg g** ^**–1**^ **)**
1	2.302	0.000
2	0.641	3.994
3	0.033	8.714

a *k*_*d*_ represents the intraparticle diffusion rate constant, and *C*_*I*_ represents the boundary-layer thickness associated with mass-transfer resistance.

### 3.4 Adsorption Isotherms

The results of the equilibrium study, as well as the adjustments of the isothermal models, are presented in Figure 6. The experimental data were analyzed using the nonlinear Langmuir, Freundlich, Redlich–Peterson, Sips, and Toth models at temperatures of 30, 40, 50, and 60 °C and concentrations of 5–500 mg L^–1^. The parameters obtained by adjusting the models are presented in Table 5.

**Figure 6:**
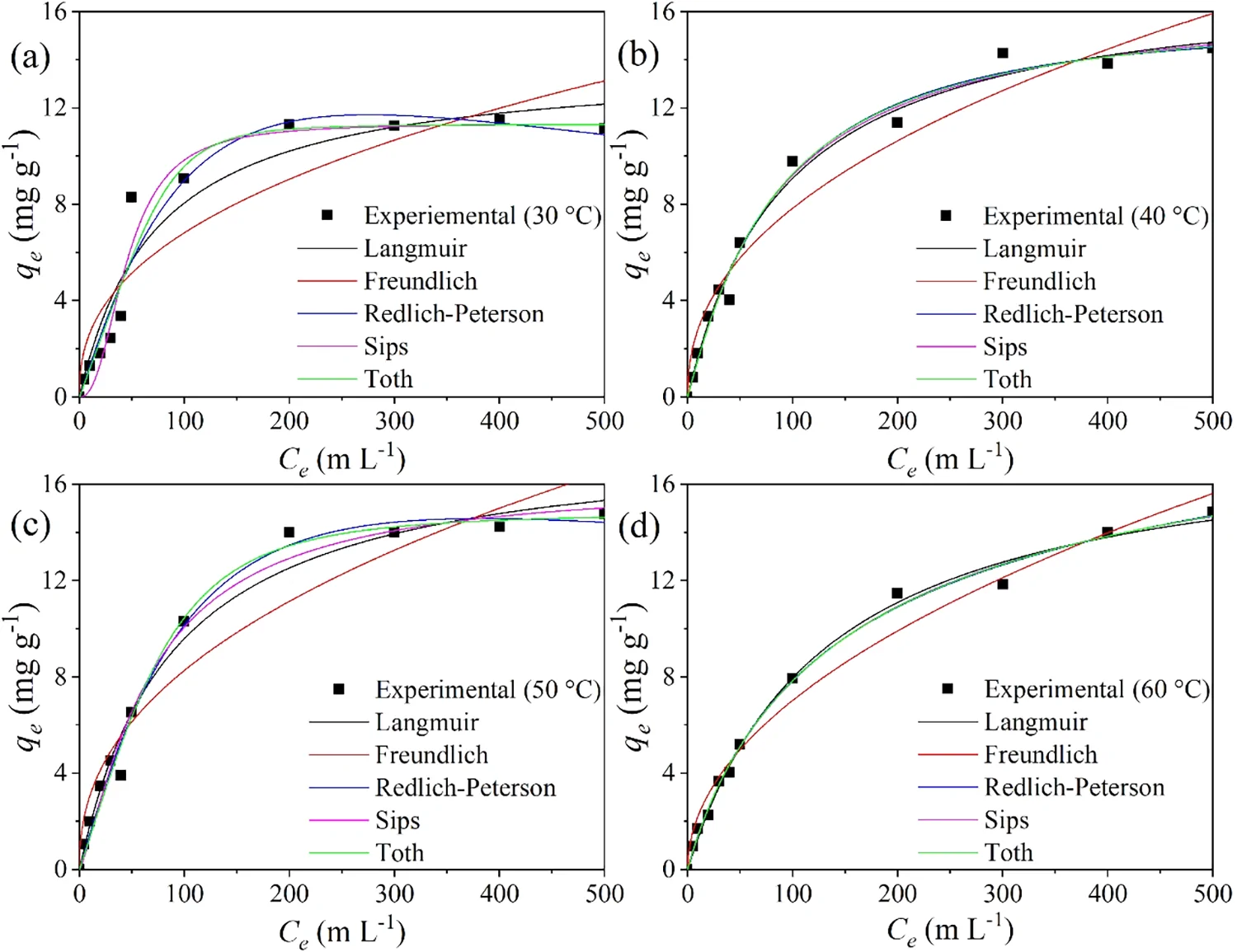
Adsorption isotherms of methylene blue onto the CDW-MC composite, fitted to the Langmuir, Freundlich, Redlich–Peterson, Sips, and Toth isotherm models at different temperatures: (a) 30 °C, (b) 40 °C, (c) 50 °C, and (d) 60 °C.

**Table 5:** Isotherm Parameters for Methylene Blue onto the CDW-MC Composite, Obtained from the Langmuir, Freundlich, Redlich–Peterson, Sips, and Toth Models at 30, 40, 50, and 60 °C^a^

**model**	**parameters**	**30 °C**	**40 °C**	**50 °C**	**60 °C**
Langmuir	*q*_max_	13.934	17.453	18.035	18.275
	*K*_*L*_	0.0137	0.0108	0.0114	0.0077
	*R* ^2^	0.9325	0.9892	0.9824	0.9950
	*x* ^2^	1.6861	0.3527	0.6299	0.1542
	ARE	2.9441	0.8228	0.9014	0.9030
	AIC	10.198	–7.012	–0.633	–16.116
Freundlich	*K*_*F*_	1.1209	1.0659	1.3636	0.5072
	*n*	2.4577	2.2677	2.3265	2.0140
	*R* ^2^	0.8563	0.9570	0.9366	0.9816
	*x* ^2^	3.5902	1.4003	2.2654	0.5723
	ARE	5.9681	3.2267	3.1076	1.8922
	AIC	18.512	8.155	13.447	–1.687
Redlich–Peterson	*K*_*R*_	0.1329	0.1712	0.1531	0.1577
	*a*_*R*_	5.486 × 10^–4^	0.0057	0.0011	0.0152
	*B*	1.4703	1.0859	1.3308	0.9110
	*R* ^2^	0.9556	0.9898	0.9901	0.9955
	*x* ^2^	1.2328	0.3705	0.3936	0.1560
	ARE	1.8413	0.8253	1.2895	0.8187
	AIC	9.523	–3.701	–3.037	–13.214
Sips	*q*_max_	11.3656	16.7932	16.2239	19.8259
	*K*_*S*_	1.164 × 10^–04^	0.0088	0.0049	0.0097
	*n*_*s*_	2.3721	1.0691	1.2626	0.9135
	*R* ^2^	0.9651	0.9895	0.9858	0.9955
	*x* ^2^	0.9699	0.3818	0.5652	0.1564
	ARE	2.9766	0.8690	1.5762	0.7377
	AIC	6.885	–3.370	0.945	–13.185
Toth	*q*_max_	11.322	16.189	14.831	20.815
	*K*_*T*_	0.0105	0.0102	0.0092	0.0079
	*n*_*T*_	3.6393	1.2153	2.2798	0.8110
	*R* ^2^	0.9604	0.9897	0.9898	0.9954
	*x* ^2^	1.1000	0.3741	0.4034	0.1577
	ARE	1.8445	0.8489	1.4718	0.7998
	AIC	8.269	–3.594	–2.765	–13.094

a *R* ^2^: coefficient of determination; *x* ^2^: chi-square statistic; ARE: average relative error; and AIC: Akaike information criterion

Figure 6 shows that the three-parameter isotherm models (Redlich–Peterson, Sips, and Toth) overlap at some points, yielding predictions and a good fit to the empirical data. However, based on statistical coefficients, the results indicate that the Toth isotherm model is the one that best fits the experimental data, presenting higher values of *R* ^2^ and lower values of *x* ^2^, AIC, and ARE. While the Toth model minimizes the deviations between the theoretical and experimental values present in the Langmuir model, the Redlich–Peterson and Sips models consist of a combination of the Freundlich and Langmuir models, tending to be simplified to the Langmuir model when applied to scenarios with low concentrations, and can be applied to homogeneous and heterogeneous systems.^42,82^ The Toth isotherm model is an extension of the Langmuir model that accounts for submonolayer adsorption and improves the accuracy of its approximation to experimental results. This model is applied to describe adsorption on heterogeneous surfaces across a wide range of adsorbate concentrations.^53^ All factors of heterogeneity of the Toth isotherm (*n*_*T*_) were not equal to unity, indicating heterogeneity in the adsorption system and corroborating material characterization. In addition, the model assumes and indicates an extended asymmetric near-Gaussian energy distribution for low adsorption energies.^67^

Table 6 presents the maximum adsorption capacities obtained in this study for CDW, MC, and CDW-MC, compared with those reported for other conventional and waste-derived adsorbents used for methylene blue removal, considering their respective operating conditions and best-fitting kinetic and isotherm models.

**Table 6:** Comparison of the Maximum Adsorption Capacity of the CDW-MC Composite with Other Methylene Blue Adsorbents Reported in the Literature

	**parameters**						
**adsorbents**	***q***_**max**_ **(mg g** ^**–1**^ **)**	***T*** **(°C)**	**pH**	***C***_***i***_ **(mg L** ^**–1**^ **)**	***t*** **(min)**	**kinetic model**	**isotherm model**
coir pith carbon^83^	5.87		6.9	40	40	PSO	Langmuir Temkin
eggshell particles^84^	0.32		12	40	15		Langmuir Freundlich
dried olive stone^85^	12.9	25	5	150	45	PSO	Temkin
olive stone activated carbon^85^	16.12	25	5	150	45	PSO	Temkin
*Citrullus colocynthis* seeds^86^	18.83	60	8	6	60	PFO	Langmuir
*Citrullus colocynthis* peels^86^	4.48	0	8	4	180	PSO	Langmuir
biochar derived from the Sesbania plant^87^	6.6		11	50	30	PSO	Langmuir
Brazil nut shells^88^	7.81	30	6.5			PSO	Langmuir
cement–carbon composite^89^	9.06				180	PSO	Langmuir
activated carbon (GAC)^89^	10.65				180	PSO	Langmuir
eco-friendly chitosan/κ-carrageenan membranes reinforced with activated bentonite^90^	18.80	50	4		200	PSO	Freundlich
walnut shell biowaste^91^	19.99	25	6.93	50	120	PSO	Langmuir
biochar prepared from the pyrolysis of mixed municipal discarded material^92^	7.2		5	100	360	PSO	
biomass fly ash self-hardened adsorbent monoliths^93^	0.66					PFO	Langmuir
cellulose acetate supported MOF-5/Crystalline Nanocellulose composite film^94^	4.29	25	11	6		PSO	Freundlich
CDW-MC (this work)	20.81	60	9	500	120	PSO	Toth

### 3.5 Adsorption Thermodynamics

The thermodynamic parameters were estimated using the van’t Hoff eq (eq S15) through the equilibrium constants obtained by the Toth model and are presented in Table 7.

**Table 7:** Thermodynamic Parameters for Methylene Blue Adsorption onto the CDW-MC Composite^a^

**temperature (°C)**	**Δ** ***G*** **°** (kJ mol^**–1**^ **)**	**Δ** ***H*** **°** (kJ mol^**–1**^ **)**	**Δ** ***S*** **°** (kJ mol^**–1**^ **K** ^**–1**^ **)**
30	–20.549	–7.693	0.042
40	–20.964		
50	–21.379		
60	–21.794		

a Δ*G°*: standard Gibbs free energy change; Δ*H°*: standard enthalpy change; Δ*S°*: standard entropy change.

Gibbs’ free energy variations are widely used to evaluate the spontaneity of a process, with Δ*G°* less than zero characterizing the reactions as exergonic and spontaneous. Thus, all of the tests carried out showed Δ*G°* negative, confirming the spontaneous nature of the adsorption. It was also observed that the additional decrease in Δ*G°* with increasing temperature indicates that higher thermal conditions favor the adsorptive process. The equilibrium constant (*K*_eq_) decreased with increasing temperature (from 8.12 to 7.83 in *ln K*_eq_ units between 30 and 60 °C) (eqs S13–S15), consistent with the negative Δ*H°* value and confirming the exothermic nature of the process. Although Δ*G°* became slightly more negative with temperature, this trend reflects the positive entropic term (−*T*Δ*S°*) rather than a more favorable adsorption equilibrium, in agreement with the considerations reported by Tran.^95^

The standard enthalpy of adsorption (Δ*H°*) allows us to infer the type of interaction involved. In general, chemisorption, which involves the formation of covalent bonds, exhibits Δ*H°* in the range of 200–800 kJ mol^–1^, while physisorption, mediated by hydrogen interactions or van der Waals forces, presents Δ*H°* below 40 kJ mol^–1^. This exothermic behavior is further corroborated by the temperature dependence of the Toth affinity constant (*K*_*T*_), which decreased from 0.0105 to 0.0079 as the temperature increased from 30 to 60 °C (Table 5). The reduction in adsorbate–adsorbent affinity with increasing temperature is fully consistent with the negative Δ*H°*, while its low magnitude confirms the predominantly physical nature of the interaction, mediated by van der Waals forces. The default positive entropy (Δ*S°*) value shows the increase in disorder at the solid–liquid interface during adsorption. Thus, the combination of Δ*G°* negative and Δ*S°* positive corroborates that the process is spontaneous, with the entropic contribution being more determinant than the enthalpic contribution to the spontaneity of adsorption.^95,96^

### 3.6 Regeneration and Reuse

The statistical significance of the main effects and their interactions on MB-saturated CDW-MC regeneration was evaluated by using ANOVA and the F-test. The results showed a high *R* ^2^ (approximately 99%) and satisfactory conditions for the F-test (Table S1), confirming the significance of the adopted model.

The Pareto chart (Figure 7a) indicates that all effects (concentration of H_2_O_2_ (*H*), concentration of Fe^2+^ (*F*), and reaction time (*T*)) and the interaction *FxT* are statistically significant (*p* ≤ 0,05). However, the largest magnitudes are observed for *F* and *T*, suggesting a greater influence on regeneration efficiency (Figure S3). It is noteworthy that despite their relatively minor magnitudes, both the interaction *FxT* and *H* were statistically significant in the construction of the quantitative model (eq S21), given the adopted confidence level.

**Figure 7:**
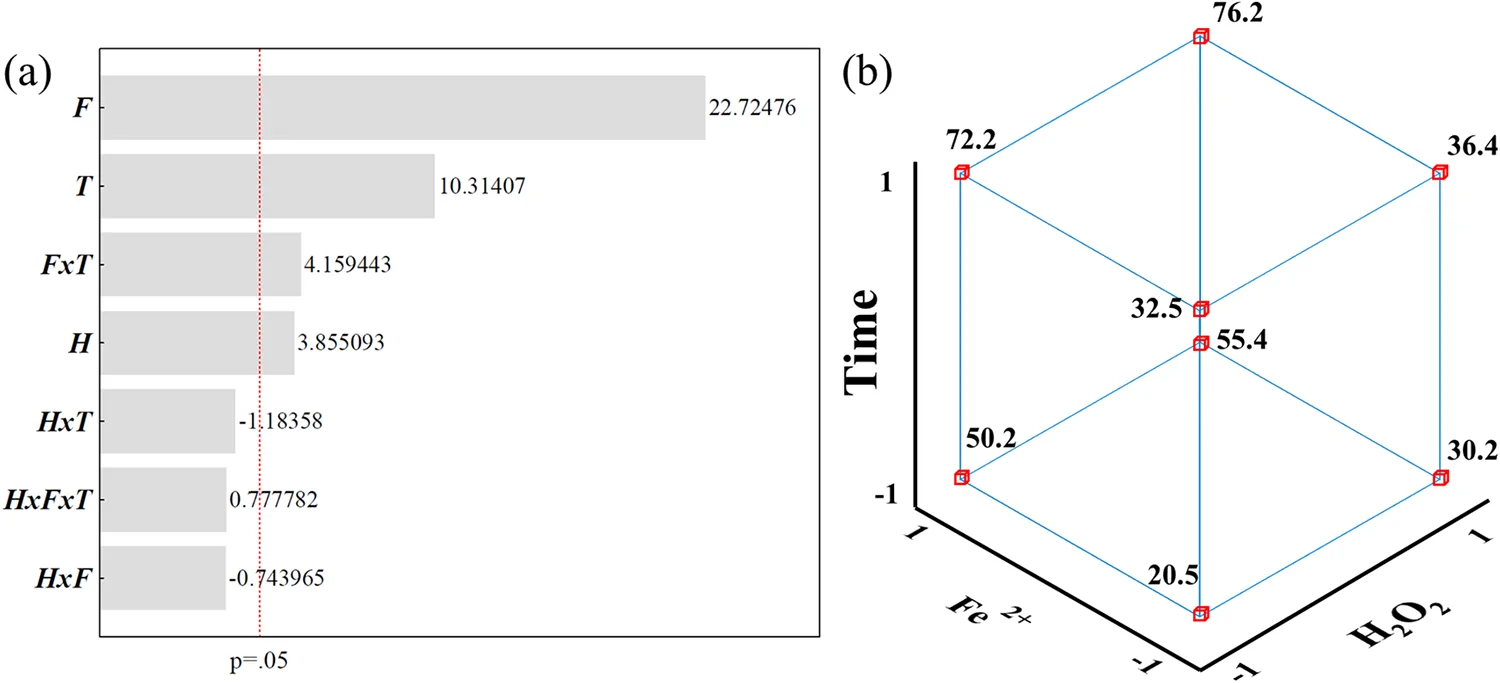
Statistical evaluation of the Fenton regeneration of methylene blue-saturated CDW-MC composite: (a) Pareto chart of the standardized effects of H_2_O_2_ concentration (*H*), Fe^2+^ concentration (*F*), contact time (*T*), and their interactions on regeneration efficiency; and (b) cubic response plot displaying the average regeneration responses obtained at the low (−1) and high (+1) coded levels of the 2^3^ factorial design.

The cubic graph of the responses shown in Figure 7b reveals a marked impact of Fe^2+^ concentration. In this graph, the edges correspond to the independent variables and their levels, while the vertices represent the average experimental results for each level combination. Increasing the Fe^2+^ concentration from 0 to 0.05 mmol L^–1^ significantly increases regeneration (Figure S3). The statistical significance of *F* in the regeneration efficiency of the composite is associated with the higher generation of ^•^OH radicals by the decomposition of H_2_O_2_ (eqs 6 and 7). Even without the addition of Fe^2+^, modest regeneration occurs, demonstrating the composite’s intrinsic catalytic activity attributed to the metal oxides (Fe_2_O_3_ and MgO) that promote the heterogeneous Fenton reaction.^58^ Even so, the catalytic activity of the spent material was considerably lower than that observed in the homogeneous Fenton process due to (i) greater availability and homogeneous distribution of Fe^2+^ ions in solution in the homogeneous system;^97^ (ii) blocking of active sites due to adsorbed MB, hindering H_2_O_2_ activation, and reducing radical generation ^•^OH;^98^ and (iii) insufficient regeneration time and low H_2_O_2_ concentration, limiting the overall efficiency of the process.

6 Fe2++H2O2→Fe3++OH•+OH−

7 $${Fe}^{3+}+H_{2}O_{2}\to {Fe}^{2+}+{HO}_{2^{•}}+H^{+}$$

Increasing the reaction time from 1 to 4 h resulted in approximately a 10% increase in regeneration. Although it is accepted that the Fenton reaction is most intense at the beginning of treatment, when H_2_O_2_, Fe^2+^ ions, and the contaminant coexist,^99^ the rapid consumption of Fe^2+^, the slow oxidation kinetics of ferric ions (eq 6), and the formation of other radicals with lower oxidation potential (eq 7) reduce the overall speed of the process.^100^ Adsorption of the dye on the solid surface makes it difficult for radicals to diffuse ^•^OH in the pores of the adsorbent by virtue of its lifetime in nanoseconds.^98^ The increase in H_2_O_2_ concentration also enhances efficiency by generating more free radicals.^10^ The highest CDW-MC regeneration efficiency, approximately 76%, was obtained at the highest levels of the variables studied (500 mmol L^–1^ H_2_O_2_, 0.5 mmol L^–1^ Fe^2+^, and 4 h reaction). These conditions were then adopted for the subsequent stages of the study.

According to the results of the preliminary investigation into the factors affecting the regeneration process, a mechanism describing the process can be formulated (Figure 8). In step 1, the regeneration process is limited by the desorption rate of the adsorbed adsorbate from the composite surface, while the degradation process occurs in solution. In this step, the homogeneous Fenton reaction predominates, as illustrated in eqs 6 and 7. In step 2, with greater availability of free sites, the oxides of the metals on the surface of the composite react with H_2_O_2_, generating radicals ^•^OH via heterogeneous Fenton-type reactions, which diffuse both in the pores of the adsorbent and in the aqueous phase, restoring active sites and increasing the efficiency of the process.

**Figure 8:**
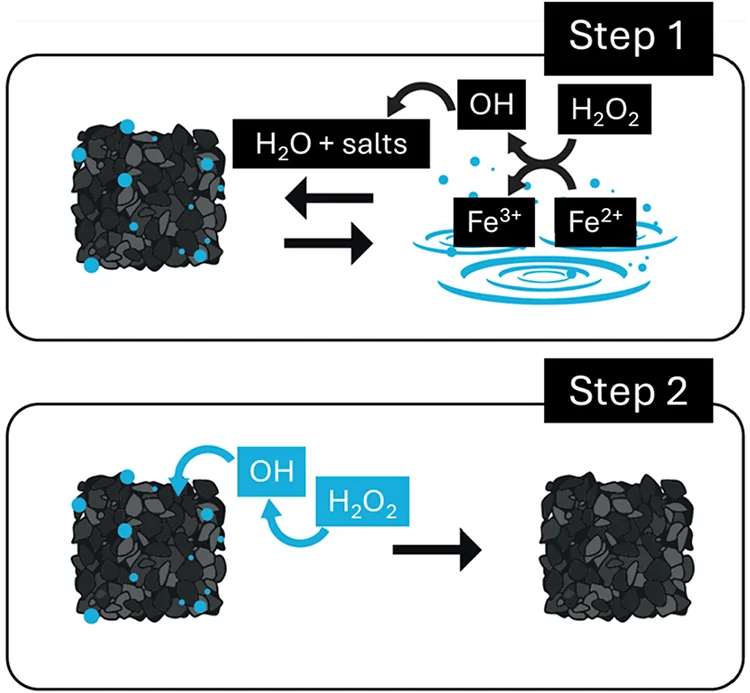
Schematic representation of the proposed two-step mechanism for the Fenton regeneration of methylene blue-saturated CDW-MC composite, involving MB desorption, homogeneous Fenton oxidation in solution, and heterogeneous Fenton-type reactions promoted by metal oxides on the composite surface.

### 3.7 Reuse of CDW-MC Composite in Regeneration–Adsorption Cycles

The adsorption capacity of the CDW-MC composite was evaluated over five consecutive adsorption–oxidation cycles under conditions that presented the best regeneration. As shown in Figure 9, the first regeneration achieved 76% efficiency, indicating the near-complete regeneration of the active sites. In cycles 2 to 4, this efficiency remained almost unchanged, indicating the stability of the Fenton reaction in the recovery of the material. Only in the fifth cycle was a slight drop in the process efficiency observed, from 76 to 70%. This decrease in efficiency, although small, may indicate blockage of composite adsorption sites by other compounds such as degradation products.^5,101^

**Figure 9:**
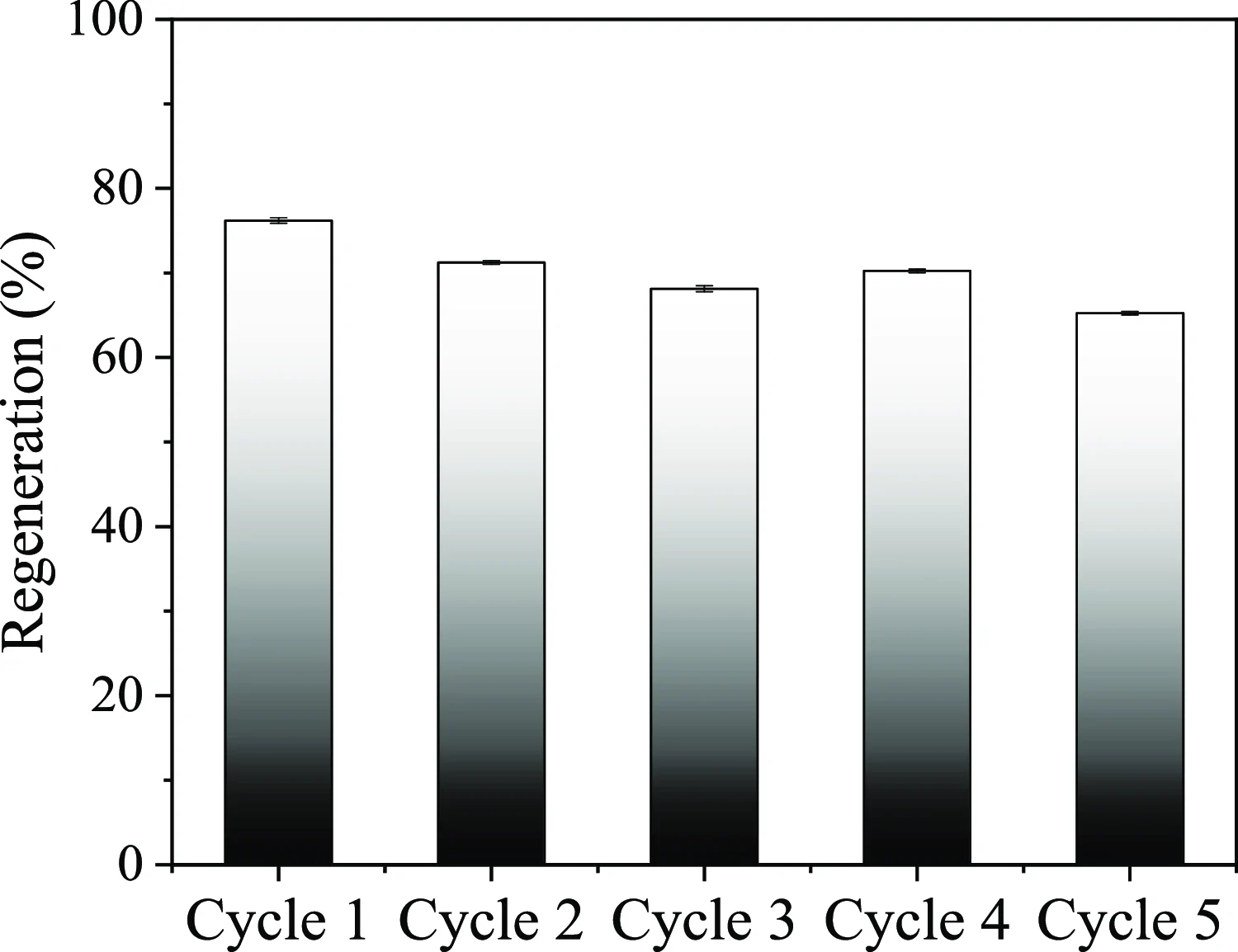
Regeneration efficiency of the methylene blue-saturated CDW-MC composite over five consecutive adsorption–Fenton regeneration cycles.

### 3.8 Limitations and Practical Considerations

CDW is an inherently heterogeneous waste stream, whose composition varies markedly with construction practices, demolition source, and region of origin.^26,27^ Consequently, the adsorption performance reported here may vary for CDW obtained from different sites or countries, and preliminary characterization of each batch is advisable before application. The present results, moreover, were obtained with methylene blue as a model cationic dye, yet CDW-derived adsorbents have also proven effective at removing other classes of contaminants, such as heavy metal ions and pharmaceuticals, in both single- and multicomponent systems,^27,28^ which suggests that the CDW-MC composite could find broader use beyond cationic dyes, although this has yet to be verified experimentally. From an environmental standpoint, although cementitious materials are known to release alkaline species into water, elemental analysis of demolition-waste leachates indicates that calcium is the only species released in appreciable amounts, with no evidence of hazardous or contaminating ones.^27^ Consistent with this, the alkaline character of the composite surface favored removal of the cationic dye in the present system, and only a modest pH increase has been reported for similar demolition-waste adsorbents under comparable condition. In terms of practical implementation, the composite was tested here in batch mode as a dispersed powder, a configuration well suited to bench-scale mechanistic studies. For larger-scale or continuous operation, immobilized formats such as fixed-bed columns are generally preferable, since they ease solid–liquid separation and allow continuous processing.^27^ Future work should therefore examine how the CDW and CDW-MC composite perform with real effluents and other pollutant classes, how they behave in continuous-flow systems, and the leaching profile and effluent quality after treatment, to consolidate their applicability within a circular-economy framework.

## 4 Conclusion

The present work demonstrated that the hybrid composite developed from construction and demolition waste (CDW) and the shells of *Mytella charruana* (MC) constitutes an innovative, sustainable, and low-cost adsorbent with remarkable performance in removing methylene blue (MB) dye from aqueous solution. The combination of the two materials produced a synergistic effect, initially reflected in a higher adsorption capacity during the screening assays (approximately 8.0 mg g^–1^) and, more significantly, in a maximum adsorption capacity of 20.82 mg g^–1^ obtained from equilibrium isotherm modeling (Toth model, 60 °C), compared to the values obtained for the individual materials. This significant improvement is attributed to the formation of a highly heterogeneous surface, with multiple active sites arising from the presence of metal oxides and carbonates.

The results showed that the adsorption process follows pseudo-second-order kinetics and provides a satisfactory fit to the Toth isotherm, suggesting heterogeneity in the system. In addition, the process proved spontaneous, exothermic, and predominantly governed by physisorption, with a positive entropic contribution, reinforcing the material’s stability and efficiency across different experimental conditions.

One of the great differentials of this study was the evaluation of the material’s regeneration, performed through sequential adsorption–oxidation cycles assisted by the Fenton process. The presence of Fe^2+^ was identified as a crucial factor for regeneration efficiency, significantly increasing the generation of hydroxyl radicals (^•^OH), thereby ensuring recovery of up to 76% of the initial adsorption capacity. It was also observed that stable performance was maintained over five cycles, with only a slight reduction in the fifth cycle, possibly due to partial site blockage caused by degradation products. These findings demonstrate not only the technical feasibility of the CDW-MC composite as an efficient adsorbent for removing organic pollutants from aqueous solutions but also its high potential for reuse, thereby reducing costs and environmental impacts. By valuing mineral waste (CDW) and biogenic waste (MC), the proposal aligns with the principles of the circular economy and the use of byproducts, aligning with global sustainability strategies.

## Supplementary Material



## Notes

Details of chemicals and reagents, as well as expanded methodological and analytical information on adsorption kinetics, isotherms, and thermodynamic parameters. It also includes the statistical evaluation of CDW-MC regeneration and reuse (ANOVA and F-tests), the corresponding supplementary table, and additional figures addressing MB removal efficiencies, the influence of pH on the composite surface, and the main effects on regeneration efficiency (PDF)
