# Supplementary material for: Hybrid Adsorbent from Construction and Mollusk Shell Wastes for Model Cationic Dye Removal: Kinetic Performance, Thermodynamic Insights, and Fenton Regenerability
Source: ACS Omega. 2026 Aug 18;11(34):51404–18. doi: 10.1021/acsomega.6c04839 (PMC13625099; doi:10.1021/acsomega.6c04839)
Supplement: Supplementary file 1 [file ao6c04839_si_001.pdf]

Hybrid Adsorbent from Construction and Mollusk Shell Wastes for Model Cationic Dye Removal: Kinetic Performance, Thermodynamic Insights, and Fenton-Regenerability

Danilo Henrique da Silva Santos[1], Larissa Farias Queiroz[1], Luiz Daniel Silva Neto[2], Keven Ewerton dos Santos[1], Denio Deivison Cerqueira Silva das Neves[1], Daniel Pinto Fernandes[1], Lucas Meili*[1]

[1]Laboratory of Processes Applied to Water Treatment, Technology Center of Federal University of Alagoas, Av. Lourival Melo Mota, s/n, Campus A.C. Simões, Tabuleiro do Martins, Maceió-AL, 57072-970, Brazil.
[2]Bioenergy and Energy Efficiency Laboratory, Institute for Technological Research, São Paulo, São Paulo, 05508-901, Brazil.

Corresponding author: Lucas Meili, Dr., lucas.meili@ctec.ufal.br, +55 (82) 999991950, Laboratory of Processes Applied to Water Treatment, Technology Center of Federal University of Alagoas, Av. Lourival de Melo Mota, s/n, Campus A. C. Simões, Tabuleiro dos Martins, Maceió-AL, 57072-970, Brazil.

S.1. Chemicals and Reagents

Methylene blue ($C_{16}H_{18}ClN_3S$), used to synthetically prepare the effluent by dissolving it in distilled water, was purchased from Synth, Brazil. Hydrochloric acid (HCl, ≥37%), ferrous sulfate ($FeSO_4$), and sodium hydroxide (NaOH, ≥96%) were obtained from Dinâmica, Brazil. Hydrogen peroxide (H_2O_2, 35%) was purchased from Neon, Brazil. All reagents used were of analytical grade.

S.2. Kinetics, Isotherms, and Thermodynamic Parameters

The kinetic study was performed with 0.1 g of CDW-MC and 20 mL of MB (pH 9 and 50 mg L^{-1}). The experiments were carried out in a finite bath (150 rpm) and at room temperature (30 °C). Samples were collected at intervals of 5, 15, 30, 45, 60, 120, 180, 240, and 300 min. The kinetic data were evaluated and fitted to the pseudo-first-order (Equation S1), pseudo-second-order (Equation S2), Elovich (Equation S3), intraparticle diffusion (Equation S4), and Boyd (Equations S5, S6, and S7) models [1].

$$q_t = q_e\,(1 - e^{-k_1 t}) \quad \text{(S1)}$$

$$q_t = \frac{q_e^2 k_2 t}{1 + q_e k_2 t} \quad \text{(S2)}$$

$$q_t = \frac{1}{\beta_E}\ln(\alpha_E \beta_E t + 1) \quad \text{(S3)}$$

$$q_t = k_d t^{1/2} + C \quad \text{(S4)}$$

$$F = 1 - \frac{6}{\pi^2}\sum_{n=1}^{\infty}\frac{1}{n^2}\,exp(-n^2 B_t) \quad \text{(S5)}$$

$$B_t = -0.497 - \ln(1 - F),\ F > 0.85 \quad \text{(S6)}$$

$$B_t = \left(\sqrt{\pi} - \sqrt{\pi - \left(\frac{\pi^2 F}{3}\right)}\right)^2,\ F < 0.85 \quad \text{(S7)}$$

where k_1, k_2 e k_d are the pseudo-first-order (min^{-1}), pseudo-second-order (g mg^{-1} min^{-1}) and intraparticle diffusion (g mg^{-1} $min^{-0,5}$) adsorption constants, respectively; α_E (mg g^{-1} min^{-1}) and β_E are Elovich constants; C is a constant related to diffusion resistance (mg g^{-1}); F is the fraction of adsorbate adsorbed at any time ($F = q_t/q_e$).

The adsorption isotherm studies were performed on 0.1 g of CDW-MC and 20 mL of MB (pH 9). The isotherms were performed at concentrations of 5, 10, 20, 30, 40, 50, 100, 200, 300, 400, and 500 mg L^{-1}, using temperatures of 30, 40, 50, and 60 ºC. The data obtained were evaluated and fitted to the Langmuir (Equation S8), Freundlich (Equation

S9), Redlich-Peterson (Equation S10), Sips (Equation S11), and Toth (Equation S12) models [2–7].

$$q_e = \frac{q_{max} K_L C_e}{1 + K_L C_e} \tag{S8}$$

$$q_e = K_F C_e^{1/n} \tag{S9}$$

$$q_e = \frac{K_R C_e}{1 + a_R C_e^B} \tag{S10}$$

$$q_e = \frac{q_{max} K_S C_e^{n_s}}{1 + K_S C_e^{n_s}} \tag{S11}$$

$$q_e = \frac{q_{max} K_T C_e}{\left(1 + (K_T C_e)^{n_T}\right)^{\frac{1}{n_T}}} \tag{S12}$$

where q_{max} is the maximum adsorption capacity of the adsorbent (mg g^{-1}); (mg g^{-1}); K_L, K_F, K_R, K_S and K_T are Langmuir (L mg^{-1}), Freundlich (mg g^{-1} (mg $L^{-1})^n$), Redlich–Peterson (L g^{-1}), Sips (L g^{-1}), and Toth (mg g^{-1}) constants of their respective isotherm models; n is the dimensionless constant representing the adsorption intensity of the Freundlich isotherm model; a_R is the constant of the Redlich–Peterson isotherm ((L $mg^{-1})^B$); B is the exponent of the Redlich–Peterson model; n_s is the exponent of the Sips isotherm model; n_T is the exponent of the Toth isotherm model, indicating the degree of heterogeneity of the surface.

The thermodynamic parameters of Gibbs free energy ($\Delta G°$), enthalpy change ($\Delta H°$), and entropy change ($\Delta S°$) of the system are determined with the best isothermal fit [8,9]. Assuming that the enthalpy and entropy changes are independent of temperature, the relationship between $\Delta G°$, $\Delta H°$, and $\Delta S°$ is expressed through Equations S13 and S14. Through the graph generated by the van't Hoff equation (Equation S15), the values of $\Delta H°$ and $\Delta S°$ are found [10,11].

$$\Delta G° = \Delta H° - T\Delta S° \tag{S13}$$

$$-RT\, ln\left(K_{eq}\right) = \Delta H° - T\Delta S° \tag{S14}$$

$$lnK_{eq} = \frac{\Delta H^{\circ}}{RT} + \frac{\Delta S^{\circ}}{R} \quad \text{(S15)}$$

where R is the gas constant (J mol K^{-1}), T is the temperature (K), and K_{eq} is the isothermal constant of adsorption.

The models were fitted by nonlinear regression, and the equation parameters were obtained using *OriginPro* software. The quality of the mathematical equation fit was assessed based on the Coefficient of Determination (R^2) (Equation S.16), Chi-square (x^2) (Equation S17), Mean Relative Error (ARE) (Equation S18) and the Akaike Information Criterion (AIC) (Equations S19 and S.20) [12,13].

$$R^2 = 1 - \frac{\sum_{i=1}^{N} (Y_{exp,i} - Y_{pre,i})^2}{\sum_{i=1}^{N} (\overline{Y_{exp,i}} - Y_{pre,i})^2} \quad \text{(S16)}$$

$$x^2 = \frac{\sum_{i=1}^{N} (Y_{exp,i} - Y_{pre,i})^2}{N-z} \quad \text{(S17)}$$

$$ARE = \sum_{i=1}^{N} \left| \frac{Y_{exp,i} - Y_{pre,i}}{Y_{exp,i}} \right| \quad \text{(S18)}$$

$$AIC = N \cdot ln\left(\frac{SSE}{N}\right) + 2z + \frac{2z(z+1)}{N-z-1} \quad \text{(S19)}$$

$$SSE = \sum_{i=1}^{N} \left(Y_{exp,i} - Y_{pre,i}\right)^2$$

(S20)

where $Y_{exp,i}$ is the experimental value, $Y_{pre,i}$ is the value predicted by the model, $\overline{Y_{exp,\,i}}$ is the average experimental value, N is the number of experimental points, and z is the number of parameters evaluated in the model.

S.3. Adsorption assays

Figure S1. Effect of particle size fraction on the methylene blue adsorption capacity of construction and demolition waste (CDW) and *Mytella charruana* shell waste (MC).

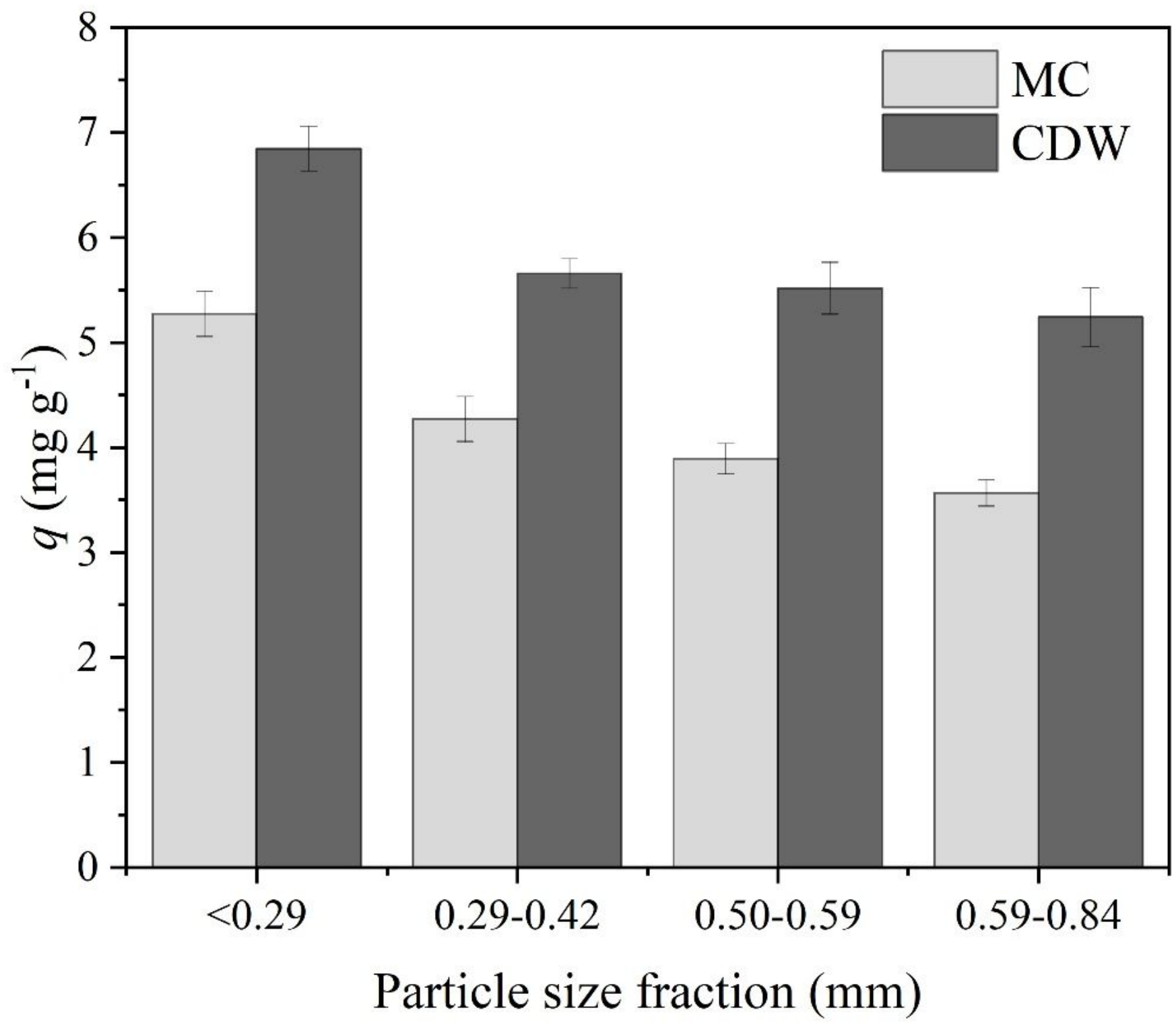

Figure S.2. Influence of pH on the composite surface.

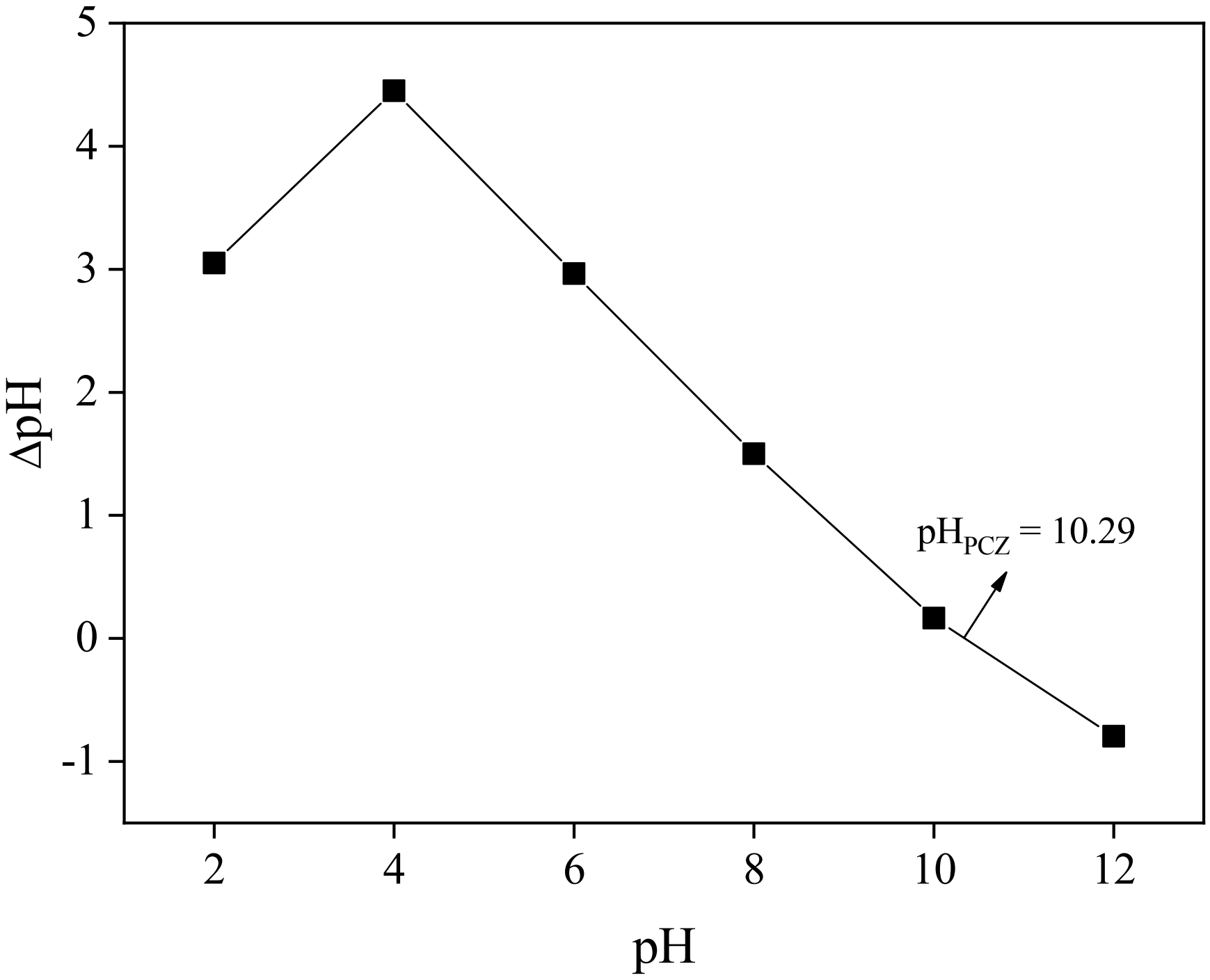

Figure S.3. Graph of the main effects on the regeneration efficiency of CDW-MC. (a) H_2O_2 concentration; (b) Fe^{2+} concentration; (c) reaction time.

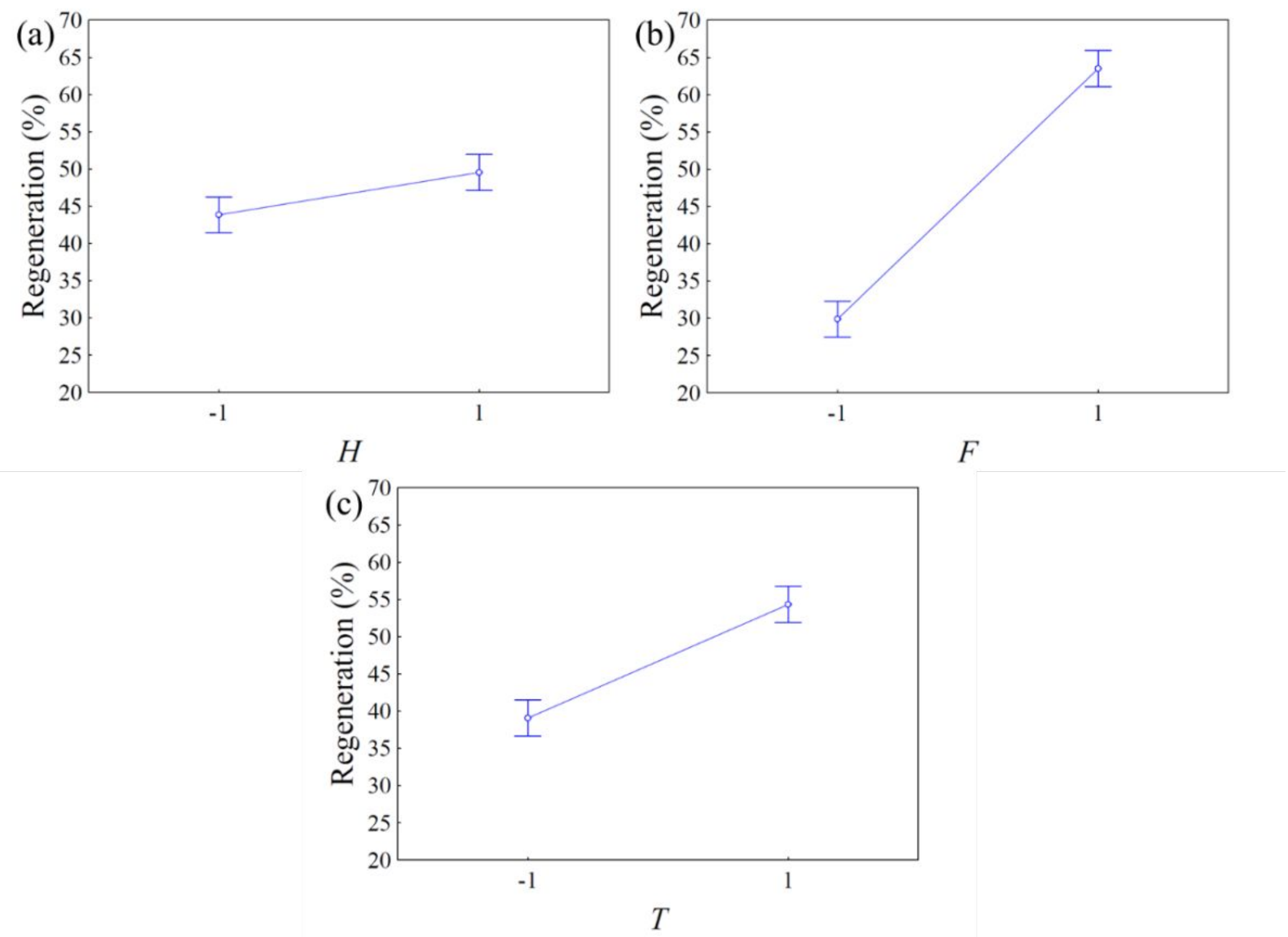

S.4. CDW-MC regeneration and reuse

The statistical significance of the main effects and their interactions in the regeneration of MB-saturated CDW-MC was evaluated using Analysis of Variance (ANOVA) and F-tests (Table S1). The results show that a high R^2 of approximately 99% was obtained, and the conditions of the F-tests were satisfied, confirming the significance of the data obtained.

Tabela S1. Analysis of Variance (ANOVA)

Parameters	Quadratic Sum	Degrees of Freedom	Mean Quadratic	Test F	$F_{tabulated}$ (95%)
Regression	5732.18	4	1433.05	180.6	3.36
Residue	87.50	11			
Lack of Adjustment	17.54	3		0.67	4.07
Pure Error	69.96	8			
Total	5907.17	15			
R^2	0.97				
R^2 max	0.9882				

$$Y = 46.7 + 5.7 \times F + 33.6 \times H + 15.25 + 6.15 \times H \times F \quad \text{(S21)}$$

where Y is the predicted response (percentage of regeneration efficiency).

Reference

(1) Karimi, S.; Tavakkoli Yaraki, M.; Karri, R. R. A Comprehensive Review of the Adsorption Mechanisms and Factors Influencing the Adsorption Process from the Perspective of Bioethanol Dehydration. *Renewable and Sustainable Energy Reviews* **2019**, *107* (July 2018), 535–553. https://doi.org/10.1016/j.rser.2019.03.025.

(2) Langmuir, I. The Adsorption of Gases on Plane Surfaces of Glass, Mica and Platinum. *J. Am. Chem. Soc.* **1918**, *40* (9), 1361–1403. https://doi.org/10.1021/ja02242a004.

(3) Freundlich, H. Over the Adsorption in Solution. *Journal of Physical Chemistry* **1906**, *57* (A), 358–471.

(4) Redlich, O.; Peterson, D. L. A Useful Adsorption Isotherm. *Journal of Physical Chemistry* **1959**, *63*, 1024.

(5) Sips, R. On the Structure of a Catalyst Surface. *J. Chem. Phys.* **1948**, *16* (5), 490–495. https://doi.org/10.1063/1.1746922.

(6) Terzyk, A. P.; Chatłas, J.; Gauden, P. A.; Rychlicki, G.; Kowalczyk, P. Developing the Solution Analogue of the Toth Adsorption Isotherm Equation. *Journal of Colloid and Interface Science*. Academic Press Inc. October 15, 2003, pp 473–476. https://doi.org/10.1016/S0021-9797(03)00569-1.

(7) Kumar, K. V.; Monteiro De Castro, M.; Martinez-Escandell, M.; Molina-Sabio, M.; Rodriguez-Reinoso, F. A Site Energy Distribution Function from Toth Isotherm for Adsorption of Gases on Heterogeneous Surfaces. *Physical Chemistry Chemical Physics* **2011**, *13* (13), 5753–5759. https://doi.org/10.1039/c0cp00902d.

(8) Budi, A.; Stipp, S. L. S.; Andersson, M. P. The Effect of Solvation and Temperature on the Adsorption of Small Organic Molecules on Calcite. *Physical Chemistry Chemical Physics* **2018**, *20* (10), 7140–7147. https://doi.org/10.1039/c7cp06747j.

(9) Ngueagni, P. T.; Woumfo, E. D.; Kumar, P. S.; Siéwé, M.; Vieillard, J.; Brun, N.; Nkuigue, P. F. Adsorption of Cu(II) Ions by Modified Horn Core: Effect of Temperature on Adsorbent Preparation and Extended Application in River Water. *J. Mol. Liq.* **2020**, *298*, 112023. https://doi.org/10.1016/j.molliq.2019.112023.

(10) Banerjee, S.; Gautam, R. K.; Rai, P.; Chattopadhyaya, M. C. Adsorptive Removal of Toxic Dyes from Aqueous Phase Using Notorious Weed Lantana Camara (Linn.) as Biosorbent. *Research on Chemical Intermediates* **2016**, *42* (6), 5677–5708. https://doi.org/10.1007/s11164-015-2397-3.

(11) Piccin, J. S.; Guterres, M.; Salau, N. P. G.; Dotto, G. L. Mass Transfer Models for the Adsorption of Acid Red 357 and Acid Black 210 by Tannery Solid Wastes. *Adsorption Science and Technology* **2017**, *35* (3–4), 300–316. https://doi.org/10.1177/0263617416675624.

(12) Gómez-de la Cruz, F. J.; Cruz-Peragón, F.; Casanova-Peláez, P. J.; Palomar-Carnicero, J. M. A Vital Stage in the Large-Scale Production of Biofuels from Spent Coffee Grounds: The Drying Kinetics. *Fuel Processing Technology* **2015**, *130* (C), 188–196. https://doi.org/10.1016/j.fuproc.2014.10.012.

(13) Nguyen, T. V. L.; Nguyen, M. D.; Nguyen, D. C.; Bach, L. G.; Lam, T. D. Model for Thin Layer Drying of Lemongrass (Cymbopogon Citratus) by Hot Air. *Processes* **2019**, *7* (1), 21. https://doi.org/10.3390/pr7010021.
